# Pegylated G-CSF Inhibits Blood Cell Depletion, Increases Platelets, Blocks Splenomegaly, and Improves Survival after Whole-Body Ionizing Irradiation but Not after Irradiation Combined with Burn

**DOI:** 10.1155/2014/481392

**Published:** 2014-03-05

**Authors:** Juliann G. Kiang, Min Zhai, Pei-Jyun Liao, David L. Bolduc, Thomas B. Elliott, Nikolai V. Gorbunov

**Affiliations:** ^1^Radiation Combined Injury Program, Armed Forces Radiobiology Research Institute, Bethesda, MD 20889, USA; ^2^Department of Radiation Biology, Uniformed Services University of the Health Sciences, Bethesda, MD 20814, USA; ^3^Department of Medicine, Uniformed Services University of the Health Sciences, Bethesda, MD 20814, USA

## Abstract

Exposure to ionizing radiation alone (radiation injury, RI) or combined with traumatic tissue injury (radiation combined injury, CI) is a crucial life-threatening factor in nuclear and radiological accidents. As demonstrated in animal models, CI results in greater mortality than RI. In our laboratory, we found that B6D2F1/J female mice exposed to ^60^Co-**γ**-photon radiation followed by 15% total-body-surface-area skin burns experienced an increment of 18% higher mortality over a 30-day observation period compared to irradiation alone; that was accompanied by severe cytopenia, thrombopenia, erythropenia, and anemia. At the 30th day after injury, neutrophils, lymphocytes, and platelets still remained very low in surviving RI and CI mice. In contrast, their RBC, hemoglobin, and hematocrit were similar to basal levels. Comparing CI and RI mice, only RI induced splenomegaly. Both RI and CI resulted in bone marrow cell depletion. It was observed that only the RI mice treated with pegylated G-CSF after RI resulted in 100% survival over the 30-day period, and pegylated G-CSF mitigated RI-induced body-weight loss and depletion of WBC and platelets. Peg-G-CSF treatment sustained RBC balance, hemoglobin levels, and hematocrits and inhibited splenomegaly after RI. The results suggest that pegylated G-CSF effectively sustained animal survival by mitigating radiation-induced cytopenia, thrombopenia, erythropenia, and anemia.

## 1. Introduction

Injuries induced by ionizing radiation alone (RI) or in combination with trauma from blast and thermal energy exposure (CI) are expected after the detonation of radiation dispersal devices or nuclear weapons. *In vivo* [1] and *in vitro* [2, 3] studies indicate that RI induced DNA double-strand breaks (DSBs), activated signal transduction pathways, elevated cytokine/chemokine concentrations in the peripheral blood, and increased systemic bacterial infection, thereby leading to cell death and multiple-organ dysfunction and failure [1, 4–6]. Traumatic injury followed by RI (i.e., CI) enhanced histopathological responses to RI, thereby increasing the mortality [1, 5–7]. Because the responses to RI and CI occur at molecular, cellular, tissue, and system levels, the complexity of the responses makes it difficult to identify countermeasures for prophylaxis, mitigation, or therapy.

RI and CI remarkably increased granulocyte colony stimulating factor (G-CSF) in mouse blood for more than 7 days [7]. The increase was initially believed to be a self-defensive response, but its appearance was probably too late to participate in repairing bone marrow damage. Bone marrow injury usually occurred within hours after RI [1, 2]. With this consideration, G-CSF and its modified form, that is, pegylated G-CSF (peg-G-CSF), have been used clinically to treat radiation-injured patients [8]. It is reported that this growth factor decreased the period of neutropenia or aplasia in the limited number of radiation accident victims studied and also enhanced neutrophil recovery following anticancer therapy [8]. The cytokine activates or primes neutrophils to enhance their function [9]. The peg-G-CSF formulation has a much longer biological half-life than G-CSF [10], thus avoiding the necessity of daily injections, which are deleterious in irradiated mice. The drug has no toxic or adverse effects in mice at the doses used. Akin to G-CSF peptide, peg-G-CSF initiates proliferation and differentiation of myeloid progenitors into mature granulocytes and induces hematopoietic stem cell mobilization from the bone marrow into the bloodstream. It is involved in recovery from infection [11, 12] and wound healing [13]. Peg-G-CSF, when combined with stem cell factor and erythropoietin, was successfully used in saving a hospital technician who had accidentally entered a ^60^Co-irradiation therapy room and received a 4.5-Gy dose of radiation [14].

This report, which is intended to stimulate interest in advancing research on peg-G-CSF in support of approval for treatment of radiation-induced neutropenia or aplasia by U.S. Food and Drug Administration, provides data from an experimental animal model designed to demonstrate the efficacy of peg-G-CSF as an effective radiomitigator.

## 2. Materials and Methods

Research was conducted in a facility accredited by the Association for Assessment and Accreditation of Laboratory Animal Care International (AAALACI). All procedures involving animals were reviewed and approved by the AFRRI Institutional Animal Care and Use Committee. Euthanasia was carried out in accordance with the recommendations and guidance of the American Veterinary Medical Association [15, 16]

### 2.1. Animals

B6D2F1/J female mice (The Jackson Laboratory, Bar Harbor, ME) were maintained in a facility accredited by the Association for Assessment and Accreditation of Laboratory Animal Care International in plastic microisolator cages on hardwood chip bedding. Commercial rodent chow and acidified tap water were provided *ad libitum* at 12 to 20 weeks of age. Animal holding rooms were maintained at 21°C ± 1°C with 50% ± 10% relative humidity using at least 10 changes/h of 100% conditioned fresh air. A 12-h 0600 (light) to 1800 (dark) full-spectrum lighting cycle was used. The AFRRI Institutional Animal Care and Use Committee approved all animal procedures.

### 2.2. Gamma Irradiation

Mice were given 9.5 Gy [1] whole-body bilateral ^60^Co gamma-photon radiation, delivered at a dose rate of 0.4 Gy/min, while held in vertically stacked, ventilated, four-compartment, and acrylic plastic boxes that provided electron equilibrium during irradiation. Empty compartments within the boxes were filled with 3-inch-long, 1-inch-diameter acrylic phantoms to ensure uniform electron scattering. The mapping of the radiation field was performed with alanine/EPR dosimetry [17] using standard alanine calibration sets from NIST and National Physical Laboratory of UK. The mapping provided dose rates to water in the core of the acrylic phantom (3 inches long, 1 inch in diameter) in each compartment of the mouse rack on the day of the mapping. The field was uniform within ±1.8% over all of the 120 compartments. The exposure time for each irradiation was determined from the mapping data; corrections for the ^60^Co decay and the small differences in the mass energy absorption coefficients for water and soft tissue were applied. The accuracy of the actual dose delivery was verified with an ionization chamber adjacent to the mouse rack, which had been calibrated in terms of dose to soft tissue in the cores of mice.

### 2.3. Skin Injury

Skin surface injuries were performed on the shaved dorsal surface of mice. Animals receiving skin burns were anesthetized by methoxyflurane inhalation. A 15% total-body-surface-area skin burn was performed within 1 h after irradiation using a 1 × 1-in custom designed template positioned centrally over the shaved dorsal skin surface. Mice received a 12-s burn from ignited 95% ethanol (0.25 mL, [5, 6]). All mice subjected to the skin injury were given 0.5 mL sterile 0.9% NaCl intraperitoneally (*i.p.*), which contained 150 mg/kg of acetaminophen (AmerisourceBergen, Glen Allen, VA) and 0.05 mg/kg of buprenorphine immediately after skin injury to alleviate pain. Four hours later, mice were given a second dose of 150 mg/kg of acetaminophen. For animals receiving skin wounds, a 15% total body-surface-area skin wound was performed within 1 h after irradiation [5, 6]. Skin-wounded mice received one dose of 150 mg/kg of acetaminophen immediately after skin injury.

### 2.4. Pegylated G-CSF

Peg-G-CSF (Neulasta; NDC: 555-13-019001) is a polyethylene glycol pharmaceutical-formulated-grade drug, also known as pegfilgrastim, and was purchased from AmerisourceBergen Corporation (Valley Forge, PA). A dose of 1000 *μ*g/kg was administered by *s.c.* injection [18, 19] in a volume of 0.2 mL 24 h, 8 d, and 15 d after RI or CI, that is, 25 *μ*g/25-g mouse. Neulasta is supplied in 0.6 mL prefilled syringes for subcutaneous injection. Each syringe contains 6 mg Peg-G-CSF in a sterile, clear, colorless, and preservative-free solution containing 0.35 mg acetate, 0.02 mg polysorbate 20, 0.02 mg sodium, and 30 mg sorbitol in water for injection, USP. Peg-G-CSF was studied in mice with sham-operation, burns, radiation, or radiation combined with burns.

### 2.5. G-CSF

G-CSF (Neupogen; Amgen, Inc., Thousand Oaks, CA, NDC: 555-13-546-10). A dose of 10 *μ*g/kg was injected *s.c.* [20] in a volume of 0.2 mL on day 1 at 24 h and thereafter once daily on days 2–14 after RI or CI. The vehicle given to control mice was sterile 0.9% sodium chloride solution for injection, USP. G-CSF was studied in mice with sham-operation, wounds, radiation, or radiation combined with wounds.

### 2.6. Antimicrobial Agents

Gentamicin sulfate cream, 0.1% (generic, E. Fougera and Co., Melville, NY, NDC 0168-007-15), was applied daily for 10 days to the skin injuries on days 1–10. Levofloxacin (LVX) (generic, Aurobindo Pharma, Ltd., Mahaboob Nagar, India, NDC 65862-537-50), 100 mg/kg in 0.2 mL/mouse, was administered *p.o.* daily for 14 days on days 3–16. Briefly, a 500-mg tablet was crushed by mortar and pestle. The LVX in the powder was dissolved in a volume of sterile water approximately one-third the total volume required to prepare the concentration needed for the average body mass of the mice to be treated. The suspension was centrifuged to remove the particulate filler and the supernatant solution was passed through a 0.45-*μ*m membrane filter into a sterile amber bottle, which was sealed with a sterile rubber stopper.

### 2.7. Survival and Body Weight

Animals were monitored at least twice daily for their general health and survival for 30 days. Their body weights were measured on days 0, 1, 3, 7, 14, 21, and 28.

### 2.8. Assessment of Blood Cell Profile in Peripheral Blood

Blood samples were collected in EDTA tubes at day 30 after RI or CI and assessed with the ADVIA 2120 Hematology System (Siemens, Deerfield, IL). Differential analysis was conducted using the peroxidase method and the light scattering techniques recommended by the manufacturer.

### 2.9. Measurements of Spleen Weights and Splenocytes

Spleens were collected from each euthanized mouse at day 30 after RI or CI. Each specimen was weighed and then homogenized in a cell strainer (BD Falcon, Bedford, MA) with 1X Hank's Balanced Salt Solution (Invitrogen, Grand Island, NY). Splenocytes in the buffer were washed with 1X ACK lysis buffer (Invitrogen) to lyse RBC, mixed by vortexing, and centrifuged at 800 ×g. Splenocytes were collected and counted using a hemocytometer.

### 2.10. Measurements of Bone Marrow Cells

Bone marrow cells from femurs were collected at day 30 after RI or CI and washed with 10 mL 1X phosphate-buffered saline (PBS). The cells were then centrifuged at 800 ×g, resuspended in 10 mL 1X PBS buffer, and then counted using a hemocytometer.

### 2.11. Statistical Analysis

Parametric data are expressed as the mean ± s.e.m. For each survival experiment, 20–22 mice per group were tested on an individual basis. Survival analyses were performed using the log-rank test. For cell analysis, one-way ANOVA, two-way ANOVA, studentized-range test, and Student's *t*-test were used for comparison of groups, with 5% as a significant level. 

## 3. Results

### 3.1. Survival and Body Weight

**Figure 1:**
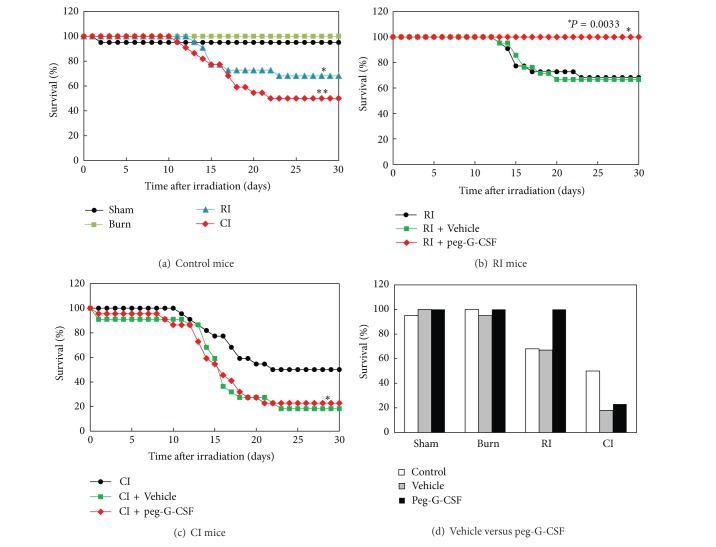
Peg-G-CSF improved survival after whole-body ionizing irradiation alone but not after irradiation combined with skin burn. *N* = 20–22 per group. For (a) *P < 0.05 versus sham, burn, and CI; **P < 0.05 versus sham burn and CI. For (b) *P = 0.0033 RI + peg-G-CSF versus RI + Vehicle and RI. For (c) *P < 0.05 versus CI. For (d) representing 100% survival in peg-G-CSF treated RI mice. RI: 9.5 Gy; CI: 9.5 Gy and skin burn.

Skin burn (15% total-body-surface area) alone did not result in mortality over a 30-day observation period (Figure 1(d)). However, skin burn following irradiation increased mortality to 50%, which was greater than mortality observed in RI mice (32%), as shown in Figure 1(a). In RI mice, vehicle treatment did not affect the radiation-induced mortality (Figures 1(b) and 1(d)). Treatment with peg-G-CSF, however, enhanced 30-day survival to 100% (Figure 1(b); *P* = 0.0033). In CI mice, both vehicle treatment and peg-G-CSF did not change the CI-induced mortality (Figures 1(c) and 1(d)).

**Figure 2:**
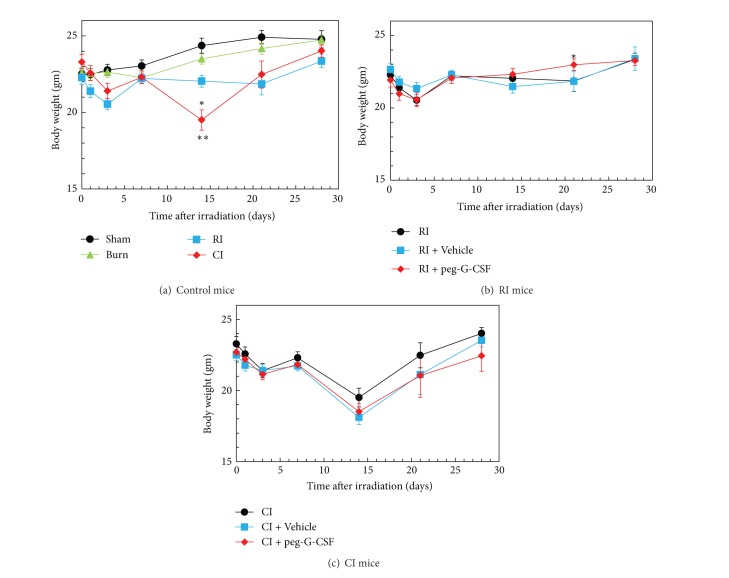
Peg-G-CSF significantly improved body-weight loss after whole-body ionizing irradiation alone but not after irradiation combined with skin burn. *N* = 20–22 per group. For (a) *P < 0.05 versus sham, burn, and CI; **P < 0.05 versus sham, burn, and RI. For (b) *P < 0.05 versus RI and RI + Vehicle. RI: 9.5 Gy; CI: 9.5 Gy and skin burn.

It is evident that RI reduced the body weight [1]. Skin burn alone did not induce body-weight loss but did enhance the radiation-induced body-weight loss (Figure 2(a)). Peg-G-CSF treatment reduced the body-weight loss in the RI mice (Figure 2(b)) but did not change body weight in the CI mice (Figure 2(c)).

**Figure 3:**
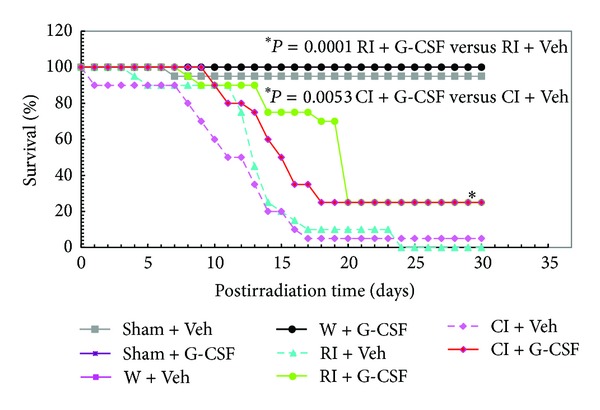
G-CSF improved survival after whole-body ionizing irradiation alone or after irradiation combined with skin wound. G-CSF was administered *s.c.* on day 1 at 24 h and thereafter once daily on days 2–14 after RI or CI. *N* = 20 per group. *P = 0.0001 RI + G-CSF versus RI + Veh; *P = 0.0053 CI + G-CSF versus CI + Veh. Veh: vehicle; RI: 9.5 Gy; CI: 9.5 Gy and skin wound.

In a separate protocol, skin wound was performed following irradiation. In this experiment, G-CSF was administered *s.c.* daily beginning on day 1 at 24 h and thereafter once daily on days 2–14. As shown in Figure 3, RI and CI control mice, which were given daily injections of vehicle for 14 days, displayed 0% and 5% survival, respectively, during the 30-day experimental period. In comparison, survival rates in RI and CI mice given G-CSF were 25% (*P* = 0.0001) and 20% (*P* = 0.0053), respectively. All nonirradiated mice survived, which were given G-CSF.

The requirement for repeated daily injection of vehicle or recombinant G-CSF for efficacy added stress to the irradiated mice, which might cause high mortality in this model of combined injury, Once-a-week administration of vehicle or peg-G-CSF reduced the total number of injections per mouse from 14 to 3, thus alleviating stress to the mice. Therefore, subsequent studies were focused on the effects of peg-G-CSF on mice receiving irradiation or in combination with burns.

### 3.2. Blood Cell Profile in Peripheral Blood

**Figure 4:**
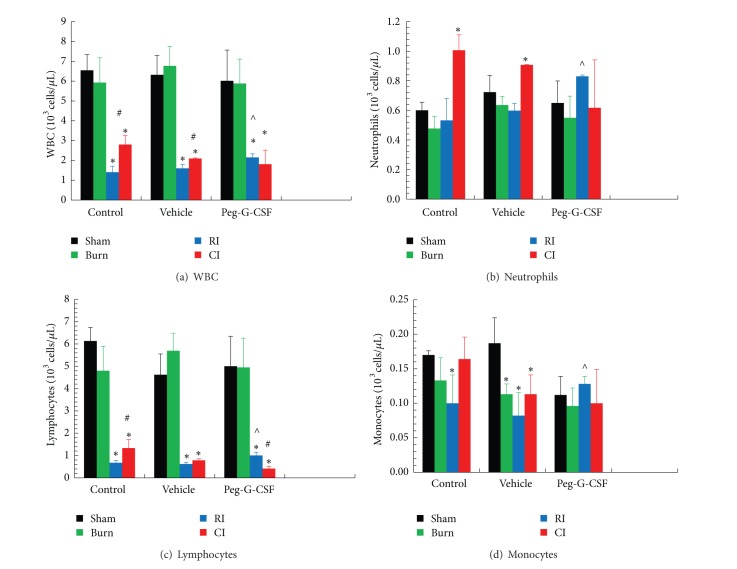
Peg-G-CSF mitigated WBC depletion after whole-body ionizing irradiation alone but not after irradiation combined with skin burn at day 30 after burn, RI, or CI. *N* = 6 per group. *P < 0.05 versus sham and burn; ^#^ *P* < 0.05 versus RI;   ^∧^ *P* < 0.05 versus RI + Vehicle. RI: 9.5 Gy; CI: 9.5 Gy and skin burn.

**Figure 5:**
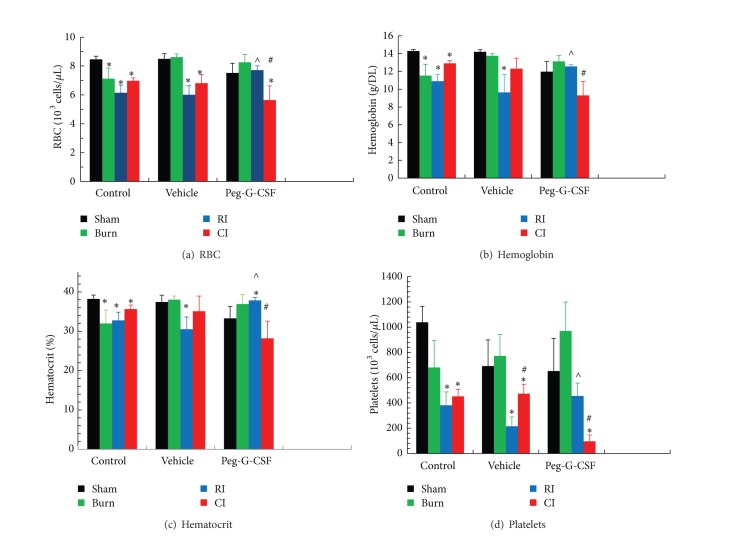
Peg-G-CSF mitigated both RBC depletion and platelet depletion after whole-body ionizing irradiation alone but not after irradiation combined with skin burn at day 30 after burn, RI, or CI. *N* = 6 per group. *P < 0.05 versus sham;   ^∧^ *P* < 0.05 versus RI + Vehicle. ^#^ *P* < 0.05 versus RI. RI: 9.5 Gy; CI: 9.5 Gy and skin burn.

RI is known to deplete WBC and RBC [1]. Skin burn alone did not affect WBC (Figure 4) but slightly decreased RBC (Figure 5) profiles. In RI mice, peg-G-CSF treatment mitigated WBC depletion slightly (Figure 4(a)), mainly numbers of neutrophils (Figure 4(b)), lymphocytes (Figure 4(c)), and monocytes (Figure 4(d)) but not in CI mice. This treatment also mitigated decreased RBC numbers (Figure 5(a)), hemoglobin (Figure 5(b)), hematocrit (Figure 5(c)), and platelets (Figure 5(d)) in RI mice.

### 3.3. Spleen Weight and Splenocytes

**Figure 6:**
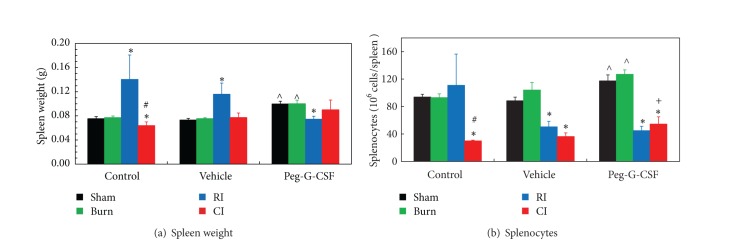
Peg-G-CSF mitigated both increased spleen weights and decreased splenocyte counts after whole-body ionizing irradiation alone and increased splenocyte counts after irradiation combined with skin burn at day 30 after burn, RI, or CI. *N* = 6 per group. *P < 0.05 versus sham and burn; ^#^ *P* < 0.05 versus RI;  ^∧^ *P* < 0.05 versus sham + Vehicle and burn + Vehicle; ^+^ *P* < 0.05 versus CI + Vehicle. RI: 9.5 Gy; CI: 9.5 Gy and skin burn.

In contrast to the effects of CI, RI alone has been shown to significantly increase spleen weight (i.e., splenomegaly) in surviving animals. Skin burn alone did not alter spleen weights and the number of splenocytes (Figure 6). Peg-G-CSF treatment increased the number of splenocytes and spleen weights in sham and burned mice but fully inhibited radiation-induced increases in spleen weight in RI mice (Figure 6(a)) and splenocyte counts below control levels (Figure 6(b)). Treatment with peg-G-CSF also decreased splenocyte counts in CI mice below control levels (Figure 6(b)).

### 3.4. Bone Marrow Cells

**Figure 7:**
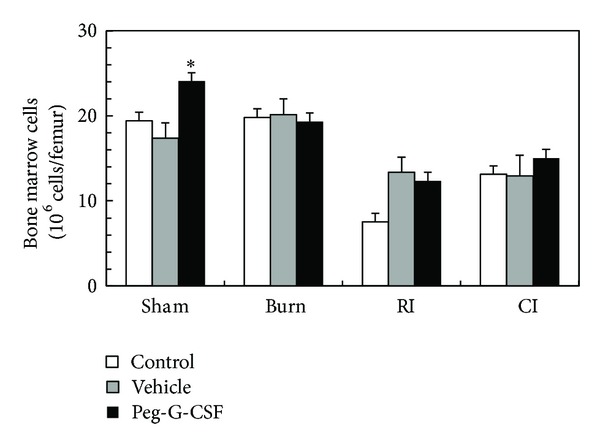
Peg-G-CSF increased bone marrow cell counts in nonirradiated control mice at day 30 after burn, RI, or CI. *N* = 6 per group. *P < 0.05 versus all other groups. RI: 9.5 Gy; CI: 9.5 Gy and skin burn.

In sham-irradiated mice, vehicle alone did not change the basal level of bone marrow cells; peg-G-CSF treatment, however, significantly elevated bone marrow cell counts (Figure 7). In skin-burned mice, neither the vehicle nor the drug treatment altered the basal level of bone marrow cell counts. Also skin burn did not change cellularity. However, in RI mice, irradiation significantly decreased the bone marrow cell count, whereas both the vehicle treatment and the drug treatment increased the cell counts, but there was no statistical difference between the vehicle treatment and the drug treatment. In CI-mice, peg-G-CSF treatment failed to improve the cellularity (Figure 7).

## 4. Discussion

This report presents data that skin burn significantly increased radiation-induced mortality and body-weight loss. The latter was thought due to injured small intestines [7]. However, the skin burn was less potent than skin wound in producing a synergistic effect with radiation in B6D2F1/J mice. These results are consistent with previous observations in rat [21, 22], guinea pig [23], dog [24], swine [25], and mice [1, 5, 6, 26–29]. Consequences of either RI or CI include acute myelosuppression, immune system inhibition, fluid imbalance, macro/microcirculation failure, massive cellular damage, and disruption of vital organ functions, which lead to multiple-organ dysfunction syndrome (MODS) and multiple-organ failure (MOF), the most frequent causes of death after irradiation [30–32].

Peg-G-CSF at the dose used displayed 100% survival in sham-operated and burned mice. Peg-G-CSF treatment enhanced 30-day survival to 100% and diminished body-weight losses after RI; this, however, was not observed in CI mice. We reported that RI and CI induced increases in G-CSF concentrations in serum (on the order of 100–1,000 pg/mL in RI mice and 2,000–10,000 pg/mL in CI mice [7]. These increases are important for recovery from RI [29, 33, 34]. In comparison, a dose of peg-G-CSF at 25 *μ*g/mouse would yield a maximum serum concentration on the order of 1,000 pg/mL [35]. Peg-G-CSF has a longer biological half-life than G-CSF [10]. Therefore, daily injections are not necessary, which would be detrimental in irradiated mice. In contrast to therapy with peg-G-CSF, our laboratory has found that *s.c.* injections of the recombinant G-CSF peptide to the same strain of irradiated mice daily for 14 days improved 30-day survival by only 25% (Figure 3). However, the recombinant G-CSF peptide was effective in improving CI mice survival by an incremental difference of 20% above the control (Figure 3). In contrast to the RI mice, peg-G-CSF failed to improve survival after CI. This could be due to the complexity of mechanisms of CI involving enhancements of serum cytokines/chemokines and systemic bacterial infection [7, 28, 29] that requires more than peg-G-CSF to manage the imbalance of homeostasis. G-CSF was also used with IL-3 to mobilize bone marrow hematopoietic progenitors to circulation [14].

RI and CI significantly reduced WBC, RBC, and platelet counts [1, 2]. At day 30 after RI or CI, surviving mice still displayed low values for WBC, mainly neutrophils and lymphocytes (Figure 4). However, the RI-induced decreases were mitigated slightly yet significantly in peg-G-CSF-treated mice. Peg-G-CSF is known to initiate proliferation and differentiation of myeloid progenitors into mature granulocytes and induce hematopoietic stem cell mobilization from the bone marrow into the bloodstream making it effective in the recovery from infection [11, 12] and wound healing [13]. Peg-G-CSF, when combined with stem cell factor and erythropoietin, was used to treat a technician, who was exposed to gamma radiation [14]. From our study, we postulate further that peg-G-CSF mobilizes hematopoietic progenitor cells in addition to myeloid cells to peripheral blood to mitigate the blood-cell depletion (Figure 5).

Peg-G-CSF treatment improved platelet counts in surviving RI-mice but not in surviving CI-mice, suggesting that this factor also can stimulate megakaryocytes in the bone marrow, similar to platelet recovery resulting from IL-12 treatment [36].

We observed that the RI mice but not CI mice exhibited splenomegaly. Splenomegaly is usually associated with disease processes that involve the destruction of abnormal RBC in the spleen. From our results, we speculate that splenomegaly may be caused by removal of RBC after irradiation. Several questions are raised. For example, how did the red and the white pulp of the spleen look like and the different cell types get distributed in the spleen? Where did injured RBC get trapped in the spleen after RI? It is also unclear why the spleen weight gained in vehicle-treated mice after RI or CI but their splenocyte counts were less than that in the sham group. Perhaps, injured RBC trapped in the spleen may increase the spleen weight but release unidentified factors that can inhibit splenocyte recovery. Further studies will be needed to address these questions.

It appears that treatment with peg-G-CSF mitigated RI-induced erythropenia and anemia. This may have been due to the drug's ability to release other cytokines that can accelerate maturation of erythroid cells in bone marrow (i.e., hematopoietic erythropoiesis) and/or in spleen (i.e., stress erythropoiesis) and to mobilize them to peripheral blood [37]. Further studies in these regards to elucidate the RI-induced splenomegaly will surely be eagerly anticipated.

It is evident that CI enhances RBC depletion, hemoglobin reduction, and hematocrit declination [1] as well as more systemic bacterial infection [4, 7]. It is not understood why CI did not induce splenomegaly but is likely associated with injury to the skin.

RI and CI result in systemic bacterial infection leading to magnified increases in cytokine concentrations in serum [7]. Acute bacterial inflammation is accompanied by excessive production of reactive oxygen and nitrogen species (ROS and RNS), which ultimately results in redox stress, a leading pathogenic factor of the septic multiple organ dysfunction syndromes [38, 39]. It is reported that in an *in vitro* study mesenchymal stromal cells survive lipopolysaccharide challenge, partially due to adaptive responses to septic oxidative stress [40]. Therefore, the possibility of survival improvement in peg-G-CSF treated RI mice mediated by adaptive responses to septic oxidative stress cannot be excluded. Additional studies are ongoing.

In summary, skin burns increased radiation-induced mortality and body-weight loss. Peg-G-CSF treatment enhanced 30-day survival to 100%, significantly mitigated body-weight loss, WBC depletion, RBC depletion, platelet depletion, and splenomegaly in RI mice. These results demonstrate efficacy of peg-G-CSF as a radiomitigator.

## Abbreviations

AFRRI: Armed Forces Radiobiology Research Institute
CI: Combined injury
*i.p.*: Intraperitoneal
*p.o.*: Oral gavage
Peg-G-CSF: Pegylated granulocyte colony stimulating factor
RBC: Red blood cells
RCI: Radiation combined injury
RI: Radiation injury
*s.c.*: Subcutaneous
TBSA: Total body surface area
VSD: Veterinary Science Department
W: Wound
WBC: White blood cells.

## Disclaimer

The views, opinions, and findings contained in this report are those of the authors and do not reflect official policy or positions of the Armed Forces Radiobiology Research Institute, the Uniformed Services University of the Health Sciences, the National Institute of Allergy and Infectious Diseases, the Department of Defense, or the United States government. The commercial products identified in this document do not imply recommendation or endorsement by the federal government and do not imply that the products identified are necessarily the best available for the purpose.
